# Increased Incidence and Clinical Picture of Childhood Narcolepsy following the 2009 H1N1 Pandemic Vaccination Campaign in Finland

**DOI:** 10.1371/journal.pone.0033723

**Published:** 2012-03-28

**Authors:** Markku Partinen, Outi Saarenpää-Heikkilä, Ismo Ilveskoski, Christer Hublin, Miika Linna, Päivi Olsén, Pekka Nokelainen, Reija Alén, Tiina Wallden, Merimaaria Espo, Harri Rusanen, Jan Olme, Heli Sätilä, Harri Arikka, Pekka Kaipainen, Ilkka Julkunen, Turkka Kirjavainen

**Affiliations:** 1 Helsinki Sleep Clinic, Finnish Narcolepsy Research Centre, Vitalmed Research Centre, Helsinki, Finland; 2 Department of Clinical Neurosciences, University of Helsinki, Helsinki, Finland; 3 Unit of Child Neurology, Department of Paediatrics, Tampere University Hospital, Tampere, Finland; 4 Department of Child Neurology, Children's Hospital, Helsinki University Central Hospital, Helsinki, Finland; 5 Finnish Institute of Occupational Health, Helsinki, Finland; 6 Department of Statistics and Registers, National Institute for Health and Welfare (THL), Helsinki, Finland; 7 Department of Child Neurology, Oulu University Hospital, Oulu, Finland; 8 Department of Child Neurology, Kuopio University Hospital, Kuopio, Finland; 9 Department of Child Neurology, Jyväskylä Central Hospital, Jyväskylä, Finland; 10 Department of Child Neurology, Central Hospital of Kymenlaakso, Kotka, Finland; 11 Department of Neurology, Oulu University Hospital, Oulu, Finland; 12 Department of Child Neurology, Vaasa Central Hospital, Vaasa, Finland; 13 Department of Child Neurology, Kanta-Häme Central Hospital, Hämeenlinna, Finland; 14 Department of Child Neurology, Turku University Hospital, Turku, Finland; 15 Rinnekoti Research Centre, Espoo, Finland; 16 Department of Vaccination and Immune Protection, National Institute for Health and Welfare (THL), Helsinki, Finland; 17 Department of Paediatrics, Children's Hospital, Helsinki University Central Hospital, Helsinki, Finland; University of Hong Kong, Hong Kong

## Abstract

**Background:**

Narcolepsy is a rare neurological sleep disorder especially in children who are younger than 10 years. In the beginning of 2010, an exceptionally large number of Finnish children suffered from an abrupt onset of excessive daytime sleepiness (EDS) and cataplexy. Therefore, we carried out a systematic analysis of the incidence of narcolepsy in Finland between the years 2002–2010.

**Methods:**

All Finnish hospitals and sleep clinics were contacted to find out the incidence of narcolepsy in 2010. The national hospital discharge register from 2002 to 2009 was used as a reference.

**Findings:**

Altogether 335 cases (all ages) of narcolepsy were diagnosed in Finland during 2002–2009 giving an annual incidence of 0.79 per 100 000 inhabitants (95% confidence interval 0.62–0.96). The average annual incidence among subjects under 17 years of age was 0.31 (0.12–0.51) per 100 000 inhabitants. In 2010, 54 children under age 17 were diagnosed with narcolepsy (5.3/100 000; 17-fold increase). Among adults ≥20 years of age the incidence rate in 2010 was 0.87/100 000, which equals that in 2002–2009. Thirty-four of the 54 children were HLA-typed, and they were all positive for narcolepsy risk allele DQB1*0602/DRB1*15. 50/54 children had received Pandemrix vaccination 0 to 242 days (median 42) before onset. All 50 had EDS with abnormal multiple sleep latency test (sleep latency <8 min and ≥2 sleep onset REM periods). The symptoms started abruptly. Forty-seven (94%) had cataplexy, which started at the same time or soon after the onset of EDS. Psychiatric symptoms were common. Otherwise the clinical picture was similar to that described in childhood narcolepsy.

**Interpretation:**

A sudden increase in the incidence of abrupt childhood narcolepsy was observed in Finland in 2010. We consider it likely that Pandemrix vaccination contributed, perhaps together with other environmental factors, to this increase in genetically susceptible children.

## Introduction

The reported prevalence of narcolepsy-cataplexy among adults in Finland is 26 cases/100 000 inhabitants (95% confidence interval CI of 0 to 60) [1]. According to Silber et al. the incidence is estimated to be 0.74 per 100 000 person-years for narcolepsy with cataplexy and 1.37 per 100 000 person-years for narcolepsy with or without cataplexy [2]. The most common symptoms of narcolepsy are unintended sleep episodes, excessive daytime sleepiness (EDS) and cataplexy [3], [4]. Most often narcolepsy starts between 12–25 years of age with the main peak of onset at 14–16 years. An onset before age of 10 years has been rare [2], [5]. Typically narcolepsy-cataplexy is characterized by the lack of hypothalamic hypocretin (orexin) production [3], [6] and strong association with HLA DR15 (DR2) and DQB1*0602 [3], [6], [7], [8]. Among Caucasians over 90% of patients with narcolepsy-cataplexy are HLA DQB1*0602 positive [3]. In a recent Danish study including a meta-analysis of seven studies the CSF-hypocretin-1 was low or undetectable in 69–100% (overall approximately 80%; 218/274) of patients with narcolepsy-cataplexy [9]. Similarly approximately 20% of patients with narcolepsy without cataplexy had low CSF hypocretin. More than 97% of patients with low CSF-hypocretin-1 are HLA DQB1*0602 –positive [9].

Genetic associations [10], [11], [12], [13], [14], [15], [16], presence of anti-tribbles 2 antibodies [17], [18], [19] and other recent observations [20], [21] suggest that autoimmune mechanisms are involved in the etiopathogenesis of narcolepsy [22], [23], [24]. Seasonality of onset [25] and reports of an association to preceding streptococcal infections [26], [27] have suggested a link with upper respiratory tract infections.

At the end of December 2009, a 7-year old boy consulted physicians because of a recent onset excessive daytime sleepiness. The H1N1 epidemic was ongoing and he had been vaccinated with Pandemrix. He had no history of recent upper respiratory tract infection or influenza-like illness (ILI). In February 2010 he was diagnosed with narcolepsy. A possible causal relationship with influenza and/or Pandemrix vaccination was suspected. Several new cases of recent onset childhood narcolepsy were observed in Pandemrix-vaccinated children, without previous history of ILI. In Finland up to fourteen diagnoses were confirmed before August 2010, bringing forward a possibility of a novel environmental trigger for increased incidence of narcolepsy in children [28]. Similar findings were reported from Sweden, and there was a time-related association observed with influenza A (H1N1) pandemic and H1N1 vaccination [28], [29], [30]. Due to the time-related association with influenza vaccination, the Finnish health authorities decided to cease Pandemrix vaccinations in August 2010 [31]. A systematic study was started to recognize all narcoleptic patients in Finland diagnosed in 2010, and to compare incidence figures with the diagnoses made between 2002 and 2009 [32]. A parallel study was focused to study the role of the AS03-adjuvanted vaccine (Pandemrix) based on register data [33]. Also in Sweden, the government funded studies were initiated [29]. Parallel case reports were reported in France and Canada, where Pandemrix or a similar AS03 adjuvanted vaccine Arepanrix was used. A possible association with H1N1 vaccination and recent onset narcolepsy was found in fourteen (9 children, 5 adults). In eleven cases Arepanrix/Pandemrix had been used (6 children <17 years and 5 adults). Two of the six children were from France, two from Canada, one from Switzerland and one from UK [34]. In the present study, we report our findings on the change in incidence of childhood narcolepsy. We also describe the clinical picture of post-Pandemrix narcolepsy in children, and compare that to previous descriptions of childhood narcolepsy.

## Methods

### Ethics statement

This study has been approved by the Institutional Review Board of the National Institute for Health and Welfare (THL). The clinical part of the study is based on case history obtained by the clinician authors. Most results are based on clinical examination of patients that was part of normal diagnostic procedures. A written informed consent was received from all patients for use of their data, and for laboratory studies and examinations that were not part of the diagnostic procedure or that were done after the diagnosis of narcolepsy had been confirmed. Parents (legal carers) signed the written informed consent on behalf of the minors/children involved in the present study.

### Collection of information of diagnosed cases of narcolepsy

In Finland the patients with specific diagnoses can be identified from the national hospital discharge registry (HILMO) kept by the National Institute for Health and Welfare (THL). This register includes comprehensively discharge abstracts from public and private patient care in hospitals and institutions in Finland. The unique personal identification code made it possible to obtain incident first-ever patient cohorts in 2002–2009 with narcolepsy with or without cataplexy (ICD-10 code G47.4) as the main diagnosis. Patients with a previously diagnosed of narcolepsy were retrospectively analysed until January 1^st^, 1997 and incident cohorts from 2002 to 2009 were included. Since there is approximately 18-month lag in the complete HILMO registry data, G47.4 diagnoses set during 2010 were collected by contacting the same sources that are used to collect data for the national registry. The included databases are based on discharge data of all hospitals and other health care organizations responsible for specific diagnosis of narcolepsy.

To verify the onset of symptoms and to gather information of the clinical picture of the diagnosed patients in 2009–2010 all available patient information was collected nation widely from hospitals and sleep centres. In addition, child neurologists in different hospitals were contacted to ensure that all patients had been recognized. The status and date of Pandemrix influenza vaccination was verified from the vaccination certificates that were filled by health care professionals. According to national guidelines all vaccinated individuals had received only one dose of Pandemrix vaccine. The presence of symptoms and their onset were obtained from medical records. They were based on face-to-face interviews with a child neurologist and the parents and children. The date of first contact with health personnel due to EDS was verified from school nurses and health care centres.

### Calculation of narcolepsy incidence rates for different years

The incidence rates for narcolepsy were calculated by dividing the number of yearly-diagnosed narcolepsy patients by the total number of people in Finland in each age group during the same year. The incidence rates in different age groups (<11, 11–16, 17–19 and people aged 20 years or more) were calculated. We used the age at the time of diagnosis to allow comparison with the data obtained from the hospital discharge registry (which includes age at diagnosis). The demographics of Finnish population were obtained from the Statistics Finland.

### Diagnosis of narcolepsy

The diagnosis of narcolepsy was based on the criteria of the International Classification of Sleep Disorders (version 2, ICSD-2) [4]. Multiple sleep latency test (MSLT) was done for all children. Analysis of cerebrospinal fluid (CSF) hypocretin-1 was done at the Rinnekoti Research Centre (Orexin A RIA kit, Phoenix Pharmaceuticals, San Mateo, CA). HLA-typing was done for some patients although it is not an official ICSD-2 diagnostic criterion. Other causes of EDS (e.g. sleep apnoea, delayed sleep phase syndrome or sleep deprivation) as well as other neurological disorders (encephalitis, encephalopathy, other neurological disorders) were excluded by polysomnography, actigraphy, thorough neurological examination, magnetic resonance imaging (MRI), EEG, CSF examinations, blood tests, and other examinations when necessary. A child neurologist always determined the diagnosis of narcolepsy. Before the patient was considered to suffer narcolepsy the diagnosis was verified by a panel of five neurologists/sleep specialists (CH, MP, OSH, PO, TK).

### Statistical analysis

Statistical analyses were performed with STATA 10.1 (Stata Corporation, TX). The incidence ratios were calculated for each year and the average incidence ratios from the period of 2002–2009 were compared to those observed in 2010 (1^st^ Jan 2010 until 31^st^ Dec 2010). The 95% confidence intervals (95% CI) are given for the average incidence rates for the years 2002–2009. Age is given as years and decimals. For statistical comparisons of the continuous variables parametric or non-parametric methods were used according to the normality of the distributions.

## Results

### Incidence of narcolepsy in 2002–2009

**Figure 1:**
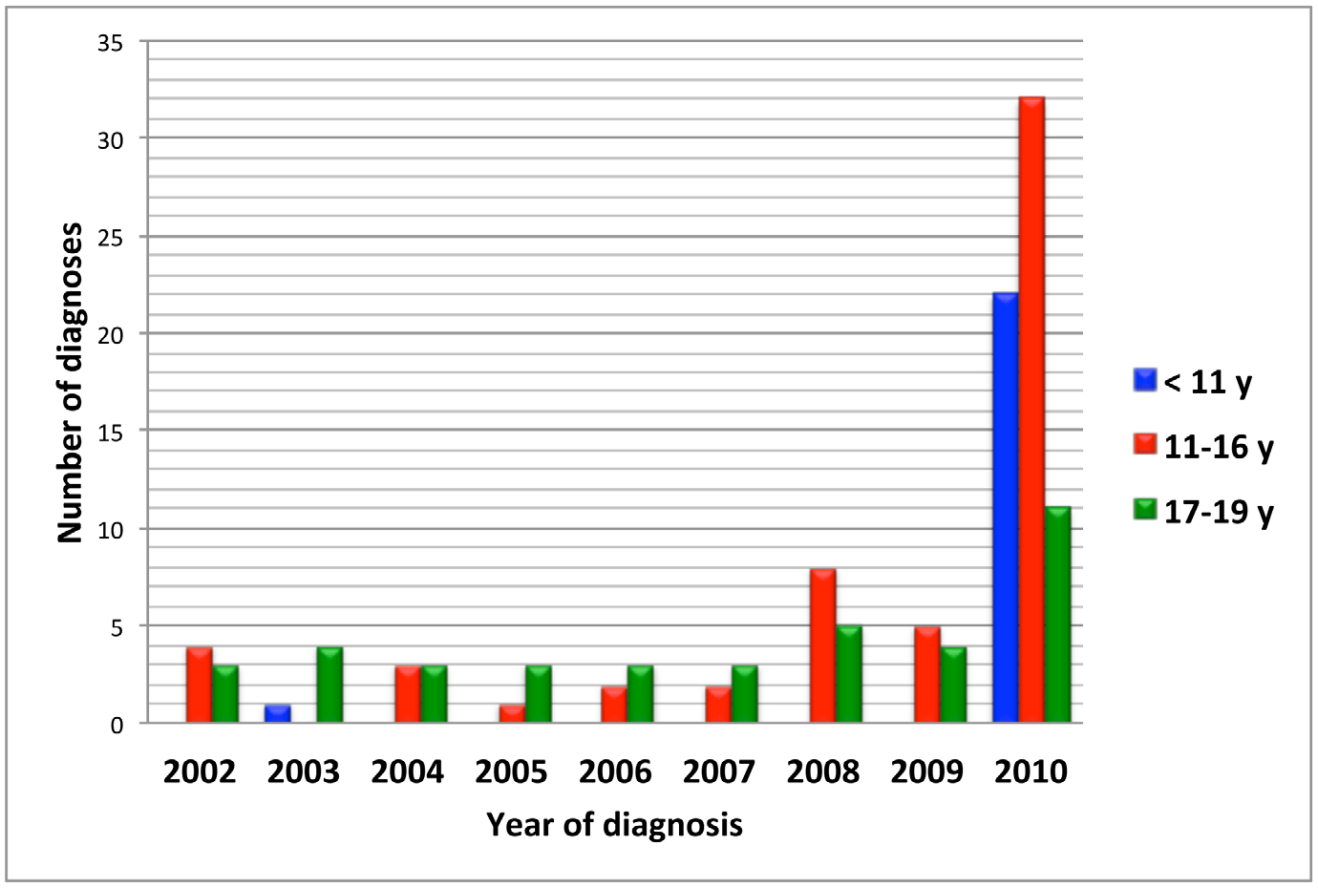
Number of new diagnoses of narcolepsy among children and adolescents aged under 20 years of age by year of diagnosis.

**Figure 2:**
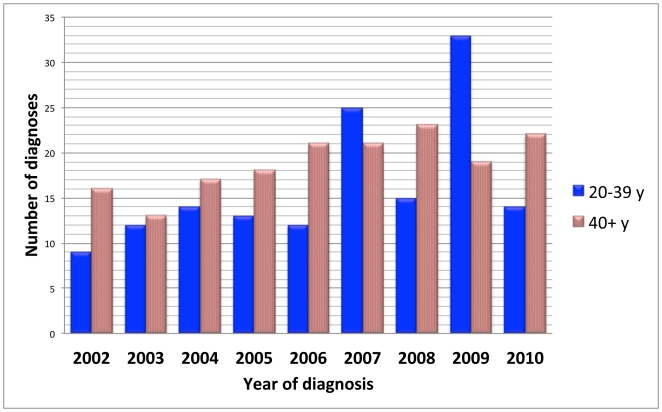
Number of new diagnoses of narcolepsy among adults aged 20 years or more by year of diagnosis.

**Figure 3:**
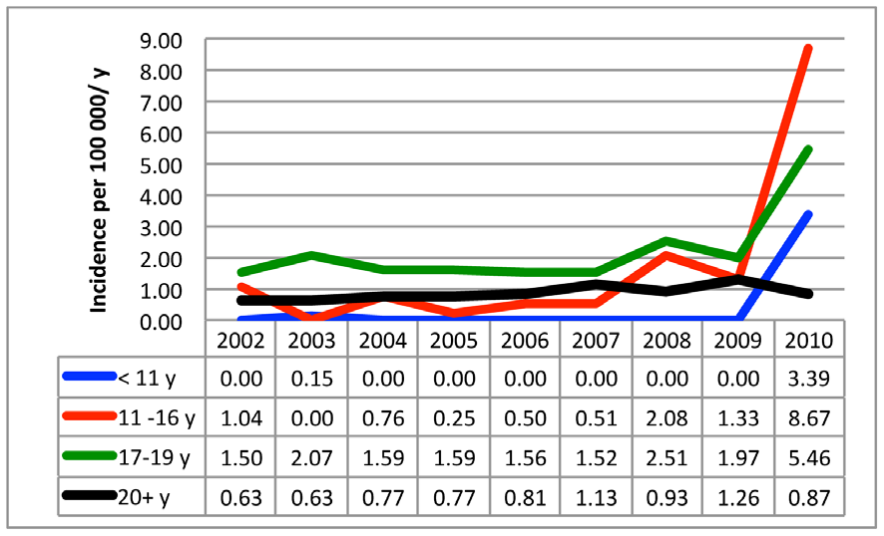
Annual incidence of narcolepsy by age group and year of diagnosis.

Altogether 335 cases of narcolepsy were diagnosed in Finland during 2002–2009 giving an annual incidence of 0.79 per 100 000 inhabitants (95% CI 0.62–0.96). Among adults ≥20 years of age, 281 new cases of narcolepsy (range 25 to 52 per year) were diagnosed between 2002–2009 giving an average annual incidence of 0.87 (95% CI 0.71–1.03) per 100 000. Twenty-eight (3 to 5 per year) cases of narcolepsy were diagnosed among individuals aged 17 to 19 with an average annual incidence of 1.79 (95% CI 1.49–2.09) per 100 000 (Figure 1, Figure 2, Figure 3, Figure S1, Figure S2). Twenty-six patients were younger than 17. In that age group the average annual incidence in 2002–2009 was 0.31 (95% CI 0.12–0.51) per 100 000 children. Only one child aged less than 11 years (a 9-year-old) was diagnosed with narcolepsy prior to 2010 (Figure 1, Figure S1).

### Incidence of narcolepsy in 2010

In 2010 altogether 101 persons were newly diagnosed for narcolepsy giving an incidence of 1.88/100 000. Of these patients 65 were younger than 20 years of age (incidence rate 5.33/100 000/year). Among adults ≥20 years of age (n = 36) the incidence rate in 2010 was 0.87/100 000, which equals with the average incidence figure in 2002–2009 (0.87). Among 17 to 19-year-olds it was 5.46/100 000 (3-fold increase). In 2010 altogether 54 cases of childhood narcolepsy were diagnosed in children and adolescents (<17 years; Appendix S1) giving an incidence rate of 5.30/100 000, which is 17 times higher than the average incidence between 2002 and 2009. In children aged <11, the incidence rate in 2010 was 3.39/100 000 (1.89 in children aged <8 and 7.56 in children aged 8 to10). Even if the highest incidence figures were seen in peripubertal and pubertal children aged 11 to 16 years (8.76 diagnoses per 100 000), the highest increase from previous figures was seen in children aged less than 11 years of age (increase from an average of 0.02 to 3.39 per 100 000 giving a 177-fold increase in incidence; see Figure 3, Figure S2).

Based on patient records 50 of the 54 children had received the pandemic H1N1 vaccine (Pandemrix, GSK) 0–242 days (median 38 days; 95% CI 40 to 67) before the onset of EDS. In 4 of the 50 vaccinated children (Appendix S1) influenza-like illness (ILI) was reported during the national H1N1 epidemic peak during the weeks 43–48 in 2009 [35]. However, laboratory confirmation of influenza infection of ILI cases had not been done. No other microbiologically confirmed infections were found in any of the 54 patients.

### Clinical picture of children (<17 years) with H1N1 vaccination-associated narcolepsy

Fifty children and adolescents, aged under 17 (age limit of paediatric care in Finland), had an onset of EDS after Pandemrix vaccination, and had a diagnosis of narcolepsy in 2010. All children were of Caucasian origin. There were 28 (56%) girls and 22 boys (Table 1). There were no significant differences in the clinical presentation of narcolepsy between boys and girls. The mean (±SD) age at the time of vaccination was 10.8±3.0 years (range 4.5 to 16.1 y). The mean age at onset was 11.0±3.1 years. The mean age at onset was 10.2±2.8 years in boys and 11.6±3.1 years in girls (P = 0.055). Eighteen per cent of the children were younger than 8 years, 24% were 8–10 years old, 38% were 11 to 13 years old and 20% were 14–16 years old at onset of symptoms. None of the 50 children had a prior history of EDS, cataplexy, or other symptoms of narcolepsy. The initial symptoms of narcolepsy included EDS, reappearance of regular daytime naps, and unintended sleep episodes (Table 1). Forty-seven of the 50 children (94%) have developed cataplexy, which started 6 to 359 days after the vaccination (median 77 days, i.e. 11 weeks). All children had abnormal multiple sleep latency test (MSLT) with a mean sleep latency of 1.8±1.4 minutes (95% CI 1.4 to 2.2) and with at least 2 (median 4; mean 3.8±0.9) sleep onset REM periods (SOREMPs). Clinically significant sleep-related breathing disturbances were not found. The mean apnea-hypopnea index was 0.4 (SD 0.4, range 0–1.4). In 11 out of 13 children CSF hypocretin-1 levels were undetectable (below 10 pg/ml) and it was pathologically low in the remaining two children (32 and 69 pg/ml; ICSD-2 criterion for narcolepsy <110 pg/ml). All three children without cataplexy had undetectable CSF hypocretin levels. MRI was done in 34 subjects. There was one arachnoidal cyst without clinical significance while in other patients MRI was normal. One child had type 1 diabetes and one had von Willebrand disease. They were both DQB1*0602 positive. Nine children (16.7%) had atopy and/or asthma and four children (7.4%) had had problems with attention and hyperactivity before onset of narcolepsy. Twenty-four (48%) children showed behavioural changes or psychiatric problems (conduct disorders/challenging and aggressive behaviour, self mutilation), which needed psychiatric treatment after the onset of narcolepsy. Psychiatric hospitalisation was necessary in four cases, and one patient needed temporarily benzodiazepine and antipsychotic treatment. No signs of central nervous system infection were found in CSF, MRI or EEG. During the spring 2010 three children received intravenous gammaglobulin without any clinical improvement. All 32 of the 50 HLA-typed children were positive for DQB1*0602/DRB1*15/DR15 – DQ6 genotype. Twenty patients were typed also for narcolepsy protective [8] genotype DQB1*0603, which was negative in all patients. At the first visit the mean body mass index (BMI) was 19.6 (SD 4.1; 95% CI 18.3–20.9; median 19.2; range 14.0–35.3) kgm^−2^. It increased by more than 5% in 26/41 (65.4%) of the children. The main clinical parameters are given in Table 1 and the Appendix S1 table.

**Table 1:** Clinical findings.

StudyCountry	Present studyFinland	Dauvilliers et al. 2010^34^Canada, France, Switzerland, UK	Aran et al. 2010^40^Italy, Israel, USA	Nevsimalova et al. 2011^41^Czech	Han et al. 2001^42^North China
**Number of subjects**	**50**	6§	51	30	29
**Period of diagnoses (yr)**	**1**	<1	2	10	1
**Proportion of females**	**28/50 (56%)**	N/A	22/51 (43%)	18/30 (60%)	8/29 (28%)
**Had Pandemrix or Arepanrix**	**50/50 (100%)**	6/6 (100%)	N/A	N/A	N/A
**Age of onset (yr), mean (SD)**	**11.0 (3.0)**	Not given	10.3 (3.57)	14.0 (3.0)***	9.2 (2.0)
**Age at onset (yr) of cataplexy, mean (SD)**	**11.4 (2.8)**	Not given	Not given	Not given	9.2 (2.0)
**Days from vaccination to onset, mean (SD); median; 95% confidence interval (days)**	**53.8 (47.1); 38; 40 to 67**	Not given	N/A	N/A	N/A
**Age (yr) at diagnosis, mean (SD)**	**11.6 (3.1)**	11.4 (4.6)	11.8 (3.57)	15.6 (3.1)***	10.7 (3.1)
**Time (yr) from onset to diagnosis**	**0.1 to 0.9 , mean 0.7 (SEM 0.03); 45 to 345 days**	<1	Mean 1.5 (SEM 0.3)	Not given	1–2
**Cataplexy**	**47/50 (94%)**	6/6 (100%)	51/51 (100%)	18/30 (60%)**	29/29 (100%)
**Time (wk) from vaccination to cataplexy,** **mean (SD); median; range**	**13.7 (10.6); 11;** **0 to 51**	6.5 (4.5)*; 4.5;3 to 15	N/A	N/A	N/A
**Months from onset of EDS to onset of cataplexy, median; range**	**0;8; 0 to 10** **In 70%≤2 (N = 47)**	−9 to 2	In 82%≤2	Not given	Cataplexy present atonset in all?
**Hypnagogic hallucinations**	**26/49 (53.1%)**	Not given	33/50 (66%)	15/30 (50%)	17/29 (59%)
**Behavioral problems**	**24/50 (48%)**	Not given	26/39 (66%)	10/30 (33.3%)	27/29 (93%)***
**Sleep paralysis**	**9/49 (18.4%)**	Not given	28/51 (55%)***	12/30 (40%)**	12/29 (41%)
**Disturbed nocturnal sleep**	**44/50 (88%)**	Not given	47/51 (92%)	Not given	Not given
**Rapid weight gain in the beginning** #	**26/41 (63.4%)** #	“frequent”	32/38 (84%)#	“frequent”	Not given
**BMI in kgm^−2^, mean (SD)**	**19.6 (4.1); (N = 42)**	Not given	25.2 (1.2)**; (N = 40)	22.7 (7.2)*; (N = 30)	20.4 (4.2); (N = 29)
**Sleep latency (min) in MSLT, mean (SD)**	**1.8 (1.4)**	Not given	2.5 (2.5)	4.0 (3.1)***	2.0 (1.3)
**SOREMPS**	**3.8 (0.9)**	Not given	Not given	3.2 (1.4)*	4.2 (0.9)*
**Short SL and ≥2 SOREMPs in MSLT**	**100% (N = 50)**	Not given	92% (N = 39)	90% (N = 30)	96.5% (N = 29)
**CSF-hypocretin-1**	**Mean (SD)** 8.6 (20.2); (N = 13)Median (range) **0 (0–69)**	Not given	Mean (SD) 4.5 (7.9); (N = 13)	<110 pg/ml ; (N = 6)	Not given

§ This table contains information of only those 6 subjects who were aged≤17 years at diagnosis. The other 10 of the 16 subjects reported by Dauvilliers et al.2010^34^ were older. They were not included to enable comparisons.

# We defined weight gain as an increase of body mass index (BMI) >5%. Aran et al^40^ defined it as >4 kg weight gain. NC: narcolepsy and cataplexy; NwC: narcolepsy without cataplexy. N/A: Not applicable; SD: standard deviation; SEM: standard error of mean. Statistically significant differences between our study and other studies are marked as.

*** for P<0.001,

** for P<0.01 and,

* for P<0.05. The numbers (N = ) in parenthesis refer to number of subjects with data.

Comparison of the present study and published studies from literature for children aged≤17 years.

To compare the clinical data of the children with onset in 2009 or 2010 (defined as POST) to those with an earlier onset (PRE) we analyzed the data of the 65 children and adolescents aged less than 20 years at diagnosis in 2010. Seven of the 65 children had an onset before 2009. The mean age at diagnosis was 15.3 years (95% CI 11.8 to 18.7) in PRE and 12.1 y (11.2 to 13.0) in POST group (P = 0.0272). The time from onset of EDS to onset of cataplexy was longer in the PRE (mean 546.2 days, 95% CI 48.7 to 1043.5) group than in the POST (mean 35.9,days, 23.3 to 48.5; P = 0.0038) group. The time from onset of symptoms to diagnosis was much longer in PRE (mean 47.6 months, 95% CI 14.2 to 80.9, range 17 to 111 months) than in POST group (mean 7.9, 95% CI 7.2 to 8.6, range 1 to 11 months; P<0.0001). There were no statistically significant differences between the groups regarding age at onset, cataplexy, aggressive behavior, or other symptoms and findings.

## Discussion

In 2010 we observed a significant increase in childhood narcolepsy in Finland. In 2002–2009, the average annual incidence of narcolepsy in children aged less than 17 years was 0.31 per 100 000 children. In 2010, the incidence rose to 5.3 per 100 000 children being about 17-fold higher as compared to previous years. In adolescents aged 17 to19, the increase was moderate (3-fold), and no increase was seen in adults ≥20 years of age. Based on registry data, a significant (6.6-fold) increase in childhood narcolepsy after Pandemrix vaccination has been reported also from Sweden [36], [37]. In a study from the Stockholm area the small number of cases of narcolepsy (six among vaccinated and two in the unvaccinated cohort) did not allow to make reliable conclusions whether clearly increased incidence of narcolepsy was found in that area [38]. In the same study the hazard ratio for Bell's palsy among vaccinated against unvaccinated was 1.25 (1.06 to 1.48). Risk for Guillain-Barré syndrome, multiple sclerosis, type 1 diabetes, and rheumatoid arthritis remained unchanged [38]. There has been no evidence of narcolepsy or other sleep-related adverse effects in recipients of MF59-adjuvanted A(H1N1) pandemic or other MF59-adjuvanted influenza vaccines [39].

In 2002 and 2009, narcolepsy was extremely rare in children aged less than 11 years. Only one 9-year-old child had been diagnosed in Finland in 2003. In 2010, twenty-two children aged less than 11 years were diagnosed giving an annual incidence of 3.39/100 000 children in that age group. In the only previous study of the incidence of narcolepsy, Silber and co-workers presented the average annual incidence of narcolepsy to be 1.37/100 000 and 0.74/100 000 for narcolepsy with cataplexy [2]. The annual incidence of narcolepsy in 2002–2009 (for all ages) in our study is 0.79 (95% CI 0.62 to 0.96) corresponding well with the published incidence rates [2].

All diagnoses were based on international criteria [4]. The symptoms were relatively severe (Table 1) but similar to those described in Caucasian children in other recent publications [34], [40], [41] and in the 1998–1999 series of 29 children from North China [42]. Psychiatric symptoms were common in our patients (48%) as it was in other studies as well (Table 1) [3], [40], [41], [42], [43]. In the Chinese study psychosocial problems were reported in 27/29 (93%) of children and academic problems were reported in 88% of the cases [42]. It is possible, however, that the psychiatric symptoms are more severe in post-vaccination narcolepsy than in “normal narcolepsy” prior to 2010. Four of our children have needed long-term psychiatric hospitalisation and antipsychotic treatment was needed in one. More studies are needed to study in more detail the nature of psychiatric symptoms relative to type of narcolepsy. One 12-year-old boy had Type 1 diabetes. He was DQB1*0602 positive, which is interesting since this HLA haplotype is regarded as protective against type 1 diabetes. Knowing the strong genetic association of narcolepsy with HLA type DQB1*0602 [3], [7], it is possible that all our narcoleptic children could be DQB1*0602 positive. Hypocretin-1 CSF levels were clearly below 110 pg/ml in all 13 children who had their CSF specimen analysed.

Fatigue and sleepiness and also more severe neurological complications have been associated with influenza [44], [45]. Symptoms of the 1918 Spanish influenza consisted also of excessive sleepiness having features similar to narcolepsy. The present 2009 pandemic H1N1 virus is genetically and immunologically more related with the Spanish influenza virus than recent seasonal influenza viruses [35], [46], [47]. The present pandemic virus has genes from avian, human and swine influenza viruses [35], [46], [47], [48] and the surface hemagglutinin (HA) and neuraminidase (NA) genes are more related to Spanish influenza genes than to present seasonal influenza A virus genes [35]. Unlike in the Spanish flu, the present pandemic 2009 H1N1 virus usually causes a mild infection and so far, except in North China [25], no narcoleptic symptoms or increased incidence of narcolepsy has been reported after the two epidemic seasons (2009/2010 and 2010/2011) of the present pandemic virus. It is anyway tempting to hypothesize that the present cases of narcolepsy could have been caused by an H1N1 influenza infection [45]. However, narcolepsy was not diagnosed in any of the nearly 8 000 laboratory confirmed H1N1 cases during the first pandemic wave in 2009 [35]. Also, none of our children had abnormal MRIs or any signs of focal encephalitis/encephalopathy that has been suggested to be involved in the destruction of the hypocretin cells of the hypothalamic area [49].

Could the increased incidence be explained by the increased awareness of narcolepsy in 2010 compared to previous years - triggered by the intensive public discussion in Finland in August 2010 of the possible association of narcolepsy with Pandemrix vaccination? This is unlikely since in the majority of our patients the symptoms of sleepiness or cataplexy started abruptly before August 2010, and the parents had consulted health care personnel already during the winter or early spring 2010. At that time there had been no news or articles of the increase in narcolepsy incidence or its possible association with the H1N1 pandemic. Also, the symptoms of narcolepsy and cataplexy were so clear that they impaired the daily life of the children. It is very unlikely that similarly severe abrupt sleepiness with cataplexy and behavioural problems would have remained unnoticed before 2009–2010. In addition, the incidence of adult and childhood narcolepsy in Finland in 2002–2009 has been similar to that seen in other countries [2], [5].

The strength of this study is that it is based on nationwide registries and thus includes the whole Finnish population. We are confident that practically all severe narcoleptic children with a fast onset in late 2009–2010 have been identified. Symptomatic narcolepsy due to brain diseases was excluded by careful neurological examination, EEG, MRI and other examinations [49].

There are, however, certain clinical aspects that have to be considered. In this study, we are limiting our analysis to the years 2002 to 2010. Some cases have remained undetected, since after 2010 we have continued to diagnose new children who have had their first symptoms during the first half of 2010 (data not presented). In most cases their initial symptoms have been less severe than in the present series, explaining why they have not been sent to specialists earlier. Thus our figure of post-Pandemrix narcolepsy cases is an underestimation. This is consistent with the reports showing that the delay from onset of symptoms to narcolepsy diagnosis may be several years [3], [43]. The increased awareness of narcolepsy in Finland, starting in the fall 2010, has lead to a clearly faster diagnosis. The incidence figures in previous years are based on nationwide hospital discharge registry data. Although the diagnostic criteria and practice in Finland has not changed, the general awareness of narcolepsy has increased, which may explain the slightly increased incidence figures in recent years. To avoid bias we compared the incidence figures from 2009 and 2010 also from the same registries in a similar fashion for all years. As stated above, the symptoms of most patients started during the spring (median 38 days after vaccination; see table 1) before any media attention took place. This means that the possible bias is limited to the speed of diagnosis and not to the disease onset itself. The onset, and the nature of symptoms and other diseases is based on information from the patients, parents, school health nurses and general practitioners, and some inadequacies are possible. In two children EDS started on the day of vaccination. They both fulfilled the diagnostic criteria of narcolepsy with cataplexy. Three children had no clear cataplexy at the moment of diagnosis, but in all of them the diagnosis was confirmed by the lack of CSF hypocretin-1. One of them has developed cataplexy during the follow-up about 18 months after onset of sleepiness. Four of the 50 post-vaccination patients were reported to suffer from ILI. However, microbiological verification of influenza or any other microbial infection had not been done suggesting a lack of clinically important microbial infections in our narcoleptic patients. Systematic determination of CSF hypocretin-1 and HLA typing were not done for all patients since they are not obligatory in the diagnosis when cataplexy is present and also MSLT is verifying narcolepsy [4]. Also some children/parents did not give their consent for CSF specimen when it was suggested.

Narcolepsy is considered an immune-mediated, autoimmune disease. In addition to a strong association especially with HLA DQB1*0602/DRB1*15/DR15 – DQ6 HLA type, narcolepsy has been associated with the presence of TRIB-2 antibodies [17], specific T-cell receptor alpha [11], and purinergic receptor P2RY11 [16] genotypes. The onset of symptoms in our children has been very abrupt which contrasts with most other autoimmune diseases [50] and with previous concepts of the natural course of narcolepsy [5]. The incidence of many autoimmune diseases has increased over the past decades [50], but the rise has never been as abrupt and as strong as in the case of childhood narcolepsy in Finland in 2010.

What could be the mechanism(s) of a sudden increase in childhood narcolepsy in Finland and Sweden? Co-occurrence is not synonymous to causation. We have therefore carefully differentiated the effects of the pandemic and the effects of the vaccination. What is remarkable in Finland is that only the Pandemrix vaccine was used and that the vaccination coverage was very high in children and adolescents (75%). In the age-group 5 to 14 years the vaccination coverage was more than 80% [33]. In children the vaccination coverage in Sweden was approximately 67% [37], [51]. In the case Pandemrix vaccination contributed to the onset of narcolepsy, the high vaccination coverage in Finland and Sweden may explain the highly increased narcolepsy incidence in these countries as compared to those countries where the H1N1 vaccination coverage was much lower, e.g. in France 10% were vaccinated, and in Italy 0.3% [51]. In the Netherlands, where only few Pandemrix-related narcolepsy cases were found, Pandemrix was used in healthy children aged 6 months to 5 years and in siblings and close relatives of children aged less than 6 months, but not in older children [52]. In addition to the mathematical explanation, the lack of an increase in narcolepsy in other countries would suggest that there are other genetic or environmental factors, in addition to the AS03 adjuvanted vaccination, contributing to the onset. Recent microbial infections have been suggested as possible environmental triggers that initiate the symptoms of narcolepsy [53]. A likely trigger in our patients could be influenza vaccination, which took place in a close time-relation with the onset of narcolepsy. Vaccination may have induced or accelerated already pre-existing autoimmunity leading to a rapid destruction of the hypocretin cells among genetically susceptible children and adolescents. All our HLA typed patients (n = 32) were of HLA DQB1*0602 type, which is the presently known major genetic susceptibility factor [54]. We cannot formally rule out the contribution of other infectious agents (H1N1, seasonal influenza, enterovirus, rhinovirus, streptococcal infection [26], or some other microbial infections) together with vaccination that could have lead to the development of narcolepsy.

A recent Chinese study observed an epidemiological link with pandemic influenza and narcolepsy, without significant relation to vaccination, showing a 3-fold increased incidence of narcolepsy 5 to 7 months after the 2009 epidemics [25]. ILI, without microbiological confirmation, was observed in only 4 of our patients (10%). There was, thus, no evidence of simultaneous or closely time-related microbial infections in most of our patients. Unfortunately, serum or CSF specimens for detailed viral and streptococcal antibody analyses were not available since there was no suspicion of microbial etiology of the narcoleptic symptoms. Almost a similar increase in the incidence of narcolepsy has been reported in Sweden, where only Pandemrix was used, as in Finland [36], [37]. Although isolated cases of narcolepsy have been diagnosed also after other vaccinations than Pandemrix, there is no evidence of an increased risk of narcolepsy with any other vaccine than the AS03 adjuvanted Pandemrix [34], [39], [55]. In order to draw a link between an environmental effect one must show that the incidence has increased significantly as compared to previous years. This was the case in the Chinese study, where the increase was not explained by Pandemrix [25]. Combining their and our results, it seems that several different or multiple triggering factors may exist for the narcolepsy to develop. Based on our study and the study by Nohynek and co-workers [33] Pandemrix vaccine was likely one of the triggers. The role of possible other closely time-related triggers remains to be studied in future epidemiological and experimental studies.

The adjuvant (AS03) in the Pandemrix vaccine is very potent, since it frequently induces local inflammatory reactions and occasional systemic side effects like fever. We can speculate that the inflammatory response was so strong that it included central nervous system affection. It is, however, difficult to say whether the systemic response produced by the adjuvant could have caused cellular damage. The hypocretin-1 levels were very low or undetectable in our narcoleptic patients, which may indicate a rapid destruction of hypocretin cells within weeks or a few months after vaccination. This does not, however, mean that the adjuvant would be causing hypocretin cell damage directly. Rather, there is a possibility that already an ongoing autoimmune process was accelerated by the nonspecific inflammatory responses induced by the vaccine or its specific components leading to the destruction of hypocretin producing cells. Thus, theoretically any inflammatory process whether it is iatrogenic or infectious (such as influenza and streptococcal infection) could non-specifically enhance the autoimmune process leading to narcolepsy. There is also a possibility that some other genetic factors, in addition to the commonly accepted HLA-DAB1*0602 risk allele [8], [11], [16], [23], [56] are enriched in Finland and Sweden making Nordic children susceptible to an acute inflammation/autoimmune-related narcolepsy.

Why were adult onset cases not increased? Theoretically it is possible that the vaccine precipitated onset in people who would have developed it later, anyway. In this case we should see a drop in adult incidence later during the coming years. Another possibility is that some children with multiple genetic predisposition factors are especially vulnerable to develop narcolepsy. In some other autoimmune diseases, such as in type 1 diabetes, early age onset is also often seen. It is also possible that the onset is more insidious in older adolescents and adults and thus there may be a delay in the diagnosis. In this case we would expect an increase in the incidence in adults during the coming years. All this mandates continued clinical and epidemiological surveillance in the future.

### Conclusions

We observed a 17-fold increase in the annual incidence of narcolepsy in 2010 as compared to previous years in children aged under 17 years of age. A common feature in the history of our 54 newly diagnosed childhood narcoleptic patients was that 50 children had received an adjuvanted pandemic influenza vaccine (Pandemrix) within 8 months before the onset of symptoms. In most cases, the development of symptoms was fast. We consider it likely that Pandemrix vaccination contributed to the increased incidence of narcolepsy in Finland in 2010 in HLA DQB1*0602 positive children. Our observations warrant further studies on the role of different environmental factors as well as pathogenetic studies to understand how a vaccination/adjuvant and other environmental triggers can cause narcolepsy.

## Supporting Information

Appendix S1 **Clinical characteristics of 54 children with narcolepsy (<17 years of age) diagnosed in 2010 in Finland.**
(DOC)

Figure S1 **Occurrence of childhood narcolepsy in 2002–2010 in Finland in different age groups.** The highest peak was seen in children aged 11 to 13 years of age. No children aged less than 8 years had been diagnosed in Finland before 2010.
(TIFF)

Figure S2 **Incidence of narcolepsy by year in different age-groups.** The highest incidences were seen in children aged from 11 to 16 years – especially in the age-group 11 to 13 years of age.
(TIFF)
