# Supplementary material for: Increased Incidence and Clinical Picture of Childhood Narcolepsy following the 2009 H1N1 Pandemic Vaccination Campaign in Finland
Source: PLoS One. 2012 Mar 28;7(3):e33723. doi: 10.1371/journal.pone.0033723 (PMC3314680; doi:10.1371/journal.pone.0033723)
Supplement: Appendix S1 — Clinical characteristics of 54 children with narcolepsy (<17 years of age) diagnosed in 2010 in Finland. (DOC) [file pone.0033723.s001.doc]

| **Appendix S1. Clinical characteristics of 54 children with narcolepsy (< 17 years of age) diagnosed in 2010 in Finland** | | | | | | | | | | | | | | | | | | | | | | |  | |  |  |
| --- | --- | --- | --- | --- | --- | --- | --- | --- | --- | --- | --- | --- | --- | --- | --- | --- | --- | --- | --- | --- | --- | --- | --- | --- | --- | --- |
| **Case** | **Sex** | **Age at dg** | **Age at vac** | **Age at onset** | **ILI** | **Vac to onset** | **Onset to dg** | **EDS** | **CPL** | **SP** | **HH** | **NM** | **DNS** | **EDS to CPL** | **Weight incr** | **MSLT SL** | **REM-onsets** | **MSLT recs** | **CSF-hcrt** | **HLA typing** | **DQB1*0603** | **Aggr beh/ psych symptoms** | |  | | |
| 1 | F | 5 | 4 | 4 | + | 55 | 237 | + | + | - | - | + | + | 304 | - | 4·25 | 4 | 4 | nd | + | nd | - | |  | | |
| 2 | F | 5 | 5 | 5 | - | 75 | 115 | + | - | - | - | + | + |  |  | 1·2 | 4 | 4 | 8·1 | DRB1*15 | nd | - | |  | | |
| 3 | M | 5 | 5 | 5 | - | 15 | 202 | + | + | - | + | + | + | 20 | + | 0·25 | 4 | 4 | nd | nd | nd | + | |  | | |
| 4 | M | 6 | 6 | 6 | - | 45 | 245 | + | + | - | - | - | + | 24 | - | 3·3 | 4 | 4 | nd | DQB1*0602 | nd | + | |  | | |
| 5 | M | 6 | 6 | 6 | - | 31 | 301 | + | - | - | - | - | - |  | - | 0·9 | 4 | 4 | 0 | DRB1*15 | nd | + | |  | | |
| 6 | M | 7 | 6 | 6 | - | 107 | 236 | + | + | - | - | + | + | 147 | - | 4·16 | 3 | 4 | nd | DR15 - DQ6 | nd | - | |  | | |
| 7 | M | 7 | 6 | 6 | - | 24 | 290 | + | + | - | + | + | + | 62 | + | 0·6 | 4 | 4 | nd | nd | nd | + | |  | | |
| 8 | M | 7 | 7 | 7 | - | 28 | 61 | + | - | - | - | - | + |  | + | 4 | 2 | 5 | 0 | DQB1*0602 | nd | - | |  | | |
| 9 | F | 8 | 7 | 7 | - | 98 | 154 | + | + | - | - | + | + | 61 | + | 0·4 | 5 | 5 | 32 | DQB1*0602 | nd | + | |  | | |
| 10 | F | 8 | 8 | 8 | - | 34 | 82 | + | + | - | + | + | + | 9 | + | 0·5 | 4 | 4 | nd | nd | nd | + | |  | | |
| 11 | M | 8 | 8 | 8 | - | 15 | 174 | + | + | - | + | + | + | 62 | + | 1 | 4 | 4 | 0 | nd | nd | + | |  | | |
| 12 | F | 8 | 8 | 8 | - | 75 | 218 | + | + | - | - | + | + | 76 | + | 0·66 | 4 | 4 | nd | DQB1*0602 | - | + | |  | | |
| 13 | M | 9 | 8 | 8 | - | 0 | 275 | + | + | - | + | + | + | 7 | + | 1·7 | 5 | 5 | nd | DQB1*0602 | - | + | |  | | |
| 14 | F | 9 | 8 | 8 | - | 32 | 335 | + | + | - | + | + | + | 45 | + | 0·38 | 4 | 4 | nd | nd | nd | + | |  | | |
| 15 | M | 9 | 9 | 9 | - | 40 | 98 | + | + | + | + | + | + | 0 | - | 2·3 | 3 | 4 | 3 | DRB1*15 | nd | + | |  | | |
| 16 | F | 9 | 9 | 9 | - | 20 | 311 | + | + | - | - | + | + | 12 | - | 1·1 | 4 | 4 | nd | DRB1*0602 | - | - | |  | | |
| 17 | F | 10 | 9 | 10 | - | 111 | 239 | + | + | - | - | + | + | 0 | + | 1·25 | 2 | 4 | nd | DR15-DQ6 | nd | + | |  | | |
| 18 | F | 11 | 10 | 10 | - | 11 | 316 | + | + | - | + | + | + | 17 |  | 1·4 | 5 | 5 | 0 | nd | nd | - | |  | | |
| 19 | M | 11 | 10 | 11 | - | 83 | 297 | + | + | - | - | - | - | 0 |  | 1·6 | 5 | 5 | 0 | DQB1*0602 | - | + | |  | | |
| 20 | F | 11 | 10 | 10 | - | 74 | 333 | + | + | + | + | + | + | 120 |  | 4 | 4 | 4 | 0 | DRB1*15 | nd | - | |  | | |
| 21 | F | 11 | 10 | 10 | - | 14 | 275 | + | + | - | + | + | + | 10 | + | 4·5 | 4 | 4 | nd | nd | nd | - | |  | | |
| 22 | M | 11 | 11 | 11 | - | 50 | 115 | + | + | - | + | + | + | 37 | + | 2·1 | 1 | 4 | nd | nd | nd | - | |  | | |
| 23 | M | 11 | 10 | 10 | - | 13 | 282 | + | + | - | - | + | + | 13 |  | 0·33 | 4 | 4 | nd | DRB1*15 | nd | - | |  | | |
| 24 | F | 11 | 11 | 11 | - | 29 | 293 | + | + | - | + | + | + | 82 | + | 2·25 | 4 | 4 | nd | nd | nd | + | |  | | |
| 25 | M | 11 | 10 | 11 | - | 113 | 260 | + | + | - | + | + | + | 9 | - | 1·1 | 3 | 5 | nd | DQB1*0602 | - | - | |  | | |
| 26 | F | 12 | 11 | 11 | + | 11 | 269 | + | + | - | - | + | + | 39 | + | 1·33 | 5 | 5 | nd | DQB1*0602 | - | - | |  | | |
| 27 | M | 12 | 11 | 11 | - | 34 | 336 | + | + | - | - | + | + | 0 |  | 2·7 | 2 | 4 | 0 | DQB1*0602 | nd | + | |  | | |
| 28 | M | 12 | 11 | 11 | - | 110 | 253 | + | + | - | - | + | + | 61 | + | 2·1 | 5 | 5 | nd | DQB1*0602 | - | + | |  | | |
| 29 | F | 12 | 11 | 11 | - | 76 | 260 | + | + | - | + | + | - | 45 | - | 0·4 | 5 | 5 | nd | DQB1*0602 | - | - | |  | | |
| 30 | F | 12 | 11 | 11 | - | 0 | 301 | + | + | - | + | + | + | 49 | + | 2·1 | 5 | 5 | nd | DQB1*0602 | - | - | |  | | |
| 31 | F | 12 | 11 | 11 | - | 34 | 250 | + | + | - | - | - | + | 0 | + | 2 | 3 | 4 | nd | nd | nd | + | |  | | |
| 32 | M | 12 | 11 | 11 | - | 6 | 269 | + | + | - | - | + | + | 0 | + | 0·35 | 4 | 4 | nd | nd | nd | + | |  | | |
| 33 | M | 12 | 11 | 11 | - | 62 | 310 | + | + | - | - | - | - | 0 | + | 0·63 | 4 | 4 | nd | nd | nd | + | |  | | |
| 34 | M | 12 | 12 | 12 | + | 67 | 45 | + | + | + | + | + | + | 9 | - | 1 | 5 | 5 | 0 | DQB1*0602 | - | + | |  | | |
| 35 | F | 13 | 12 | 13 | - | 242 | 159 | + | + | - | - | + | + | -62 | + | 3 | 4 | 4 | nd | DQB1*0602 | nd | + | |  | | |
| 36 | M | 13 | 12 | 12 | - | 56 | 310 | + | + | + | + | - | - | 33 | + | 0·75 | 4 | 4 | nd | DQB1*0602 | - | + | |  | | |
| 37 | F | 13 | 12 | 12 | - | 61 | 328 | + | + | + | + | - | + | 15 |  | 2·88 | 3 | 4 | nd | nd | nd | - | |  | | |
| 38 | M | 13 | 12 | 12 | - | 104 | 190 | + | + | - | - | - | - | 108 | + | 0·25 | 4 | 4 | nd | DRB1*15 | nd | - | |  | | |
| 39 | F | 14 | 12 | 13 | - | 76 | 299 | + | + | - | - |  | + | 90 |  | 0·3 | 5 | 5 | nd | nd | nd | - | |  | | |
| 40 | F | 14 | 13 | 13 | - | 33 | 345 | + | + | - | - | + | + | 62 | + | 3 | 3 | 5 | nd | DQB1*0602 | - | - | |  | | |
| 41 | F | 14 | 13 | 14 | - | 96 | 242 | + | + | - | + | - | + | 0 | + | 0·88 | 4 | 4 | nd | nd | nd | - | |  | | |
| 42 | F | 14 | 14 | 14 | - | 36 | 147 | + | + | + | + | + | + | 31 | - | 1·25 | 4 | 4 | nd | nd | nd | - | |  | | |
| 43 | M | 14 | 14 | 14 | - | 39 | 284 | + | + | - | - | + | + | 146 | + | 2·2 | 3 | 5 | nd | DQB1*0602 | - | - | |  | | |
| 44 | F | 15 | 15 | 15 | + | 25 | 153 | + | + |  |  |  | + | 27 | - | 3·6 | 2 | 4 | 69 | DQB1*0602 | - | - | |  | | |
| 45 | F | 15 | 15 | 15 | - | 31 | 180 | + | + | - | + | - | + | 0 | - | 1·24 | 4 | 4 | nd | DQB1*0602 | - | + | |  | | |
| 46 | F | 15 | 14 | 14 | - | 28 | 320 | + | + | + | + | - | + | 73 | - | 0·6 | 2 | 4 | nd | DR15-DQ6 | nd | - | |  | | |
| 47 | F | 15 | 14 | 15 | - | 197 | 163 | + | + | - | + | + | + | 0 | - | 6·3 | 3 | 4 | nd | nd | nd | + | |  | | |
| 48 | M | 16 | 15 | 15 | - | 14 | 281 | + | + | + | + | - | + | 44 | + | 3·25 | 4 | 4 | 0 | DR15 DQ6 | nd | - | |  | | |
| 49 | F | 16 | 15 | 15 | - | 48 | 246 | + | + | - | + | - | + | 15 |  | 1·5 | 4 | 4 | nd | nd | nd | - | |  | | |
| 50 | F | 16 | 16 | 16 | - | 11 | 310 | + | + | + | + | + | + | 0 | - | 2 | 4 | 4 | nd | DQB1*0602 | - | - | |  | | |
| 51 | M | 9 | 8 | 7 | - | -410 | 799 | + | + | - | - | + | + | 700 | + | 0·38 | 4 | 4 | nd | nd | nd | + | |  | | |
| 52 | F | 11 | 10 | 9 | - | -358 | 655 | + | + | - | - | - | + | 396 | + | 0·37 | 4 | 4 | nd | DQB1*0602 | - | + | |  | | |
| 53 | M | 13 | 12 | 4 | - | -2944 | 3328 | + | + | - | - | + | + | 0 |  | 4·5 | 1 | 4 | 61 | DRB1*15 | nd | + | |  | | |
| 54 | M | 16 | 16 | 15 | - | -155 | 196 | + | + | - | - |  | + | 0 |  | 0·8 | 4 | 4 | nd | nd | nd | - | |  | | |
| Cases 1 to 50 were associated to H1N1 vaccination. The symptoms of the cases 51 to 54 started before the H1N1 epidemic and vaccination campaign. ILI: influenza  like illness; EDS: excessive daytime sleepiness; CPL: cataplexy; SP: sleep paralysis; HH: hypnagogic hallucinations; NM: nightmares; DNS: disturbed nocturnal sleep; | | | | | | | | | | | | | | | | | | | | | | | | | | |
| EDS to CPL: time (days) from onset of EDS to onset of CPL; Weight incr: Increase of body mass index (BMI) by more than 5% during the first 6-12 months; MSLT SL: | | | | | | | | | | | | | | | | | | | | | | | | | | |
| mean sleep latency in the Multiple Sleep Latency Test (minutes); REM-onsets: number of REM-onset periods in MSLT; MSLT recs: number of MSLT recordings;  CSF-hcrt: CSF-hypocretin-1 in pg/ml; Aggr beh/ psych symptoms: Presence of aggressive behavior/ challenging behavior; MPH: methylphenidate; +: yes/present;  -: no/not present; nd: not done. | | | | | | | | | | | | | | | | | | | | | | | | | | |
| iM | | | | | | | | | | | | | | | | | | | | | | | | | | |
|  | | | | | | | | | | | | | | | | | | | | | | | | | | |
