# Supplementary figures and images for: Increased Incidence and Clinical Picture of Childhood Narcolepsy following the 2009 H1N1 Pandemic Vaccination Campaign in Finland
Source: PLoS One. 2012 Mar 28;7(3):e33723. doi: 10.1371/journal.pone.0033723 (PMC3314680; doi:10.1371/journal.pone.0033723)

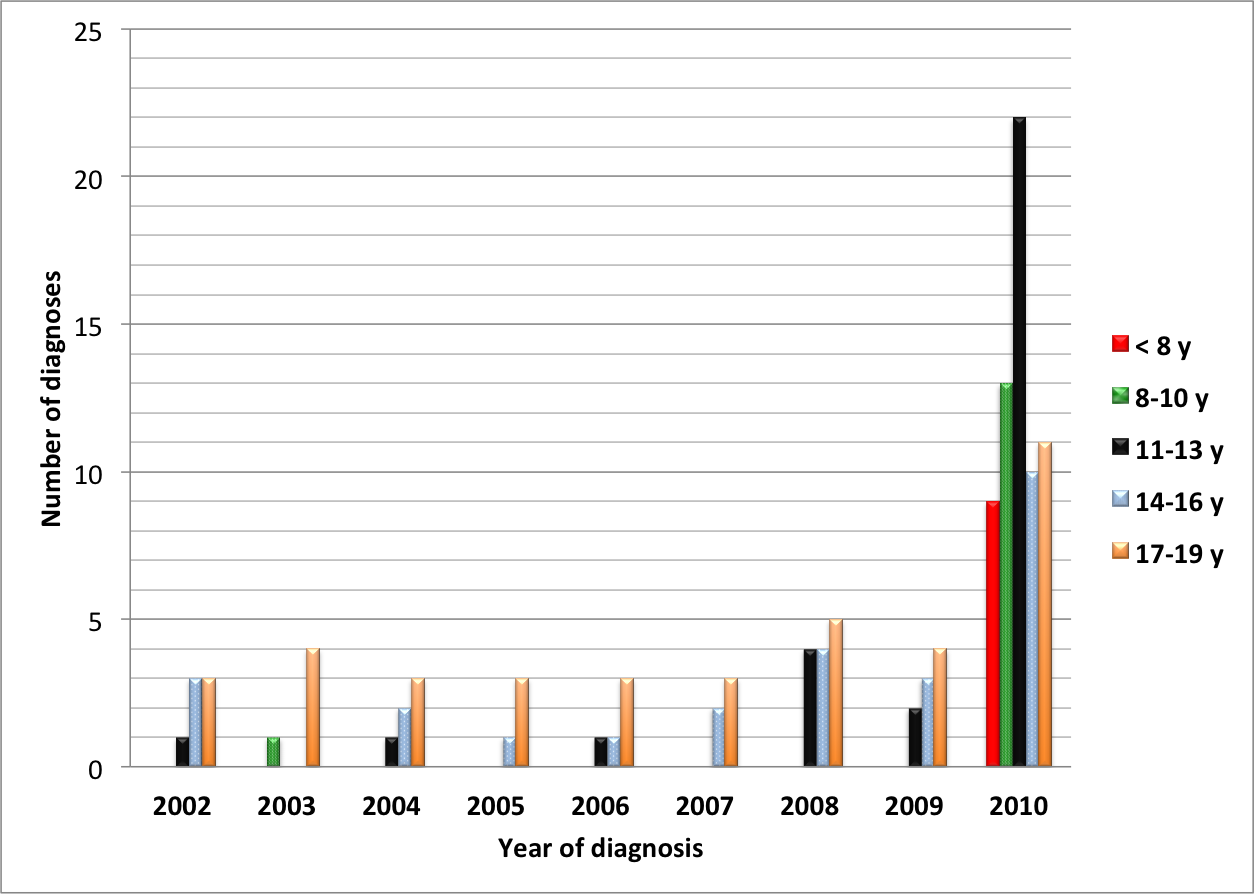

Supplement: Figure S1 — Occurrence of childhood narcolepsy in 2002–2010 in Finland in different age groups. The highest peak was seen in children aged 11 to 13 years of age. No children aged less than 8 years had been diagnosed in Finland before 2010. (TIFF) [file pone.0033723.s002.tif]

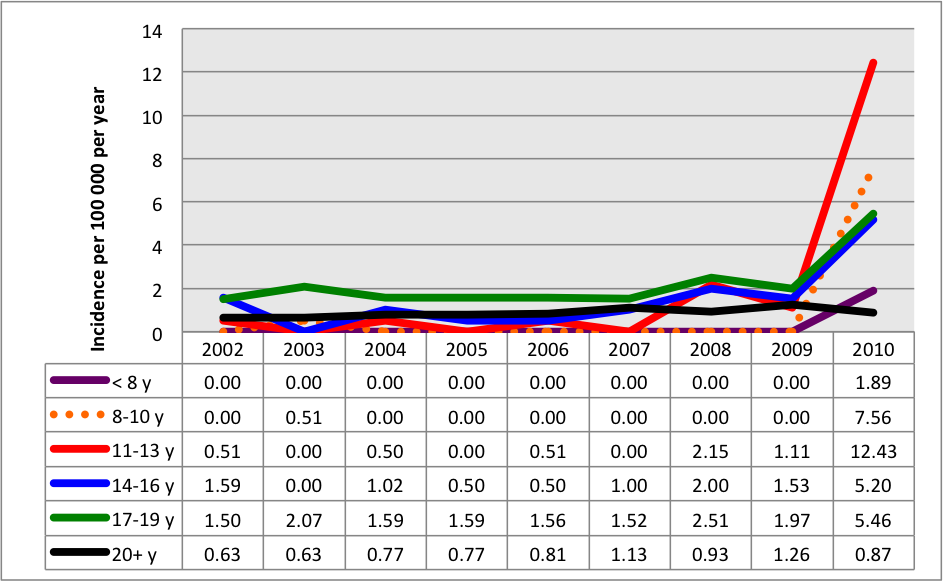

Supplement: Figure S2 — Incidence of narcolepsy by year in different age-groups. The highest incidences were seen in children aged from 11 to 16 years – especially in the age-group 11 to 13 years of age. (TIFF) [file pone.0033723.s003.tif]
